# *Yersinia pseudotuberculosis* O:1 Traced to Raw Carrots, Finland 

**DOI:** 10.3201/eid1412.080284

**Published:** 2008-12

**Authors:** Susanna Kangas, Johanna Takkinen, Marjaana Hakkinen, Ulla-Maija Nakari, Tuula Johansson, Heikki Henttonen, Laura Virtaluoto, Anja Siitonen, Jukka Ollgren, Markku Kuusi

**Affiliations:** Helsinki University, Helsinki, Finland (S. Kangas); National Public Health Institute, Helsinki (S. Kangas, J. Takkinen, U.-M. Nakari, A. Siitonen, J. Ollgen, M. Kuusi); Finnish Food Safety Authority, Helsinki (M. Hakkinen, T. Johansson); Finnish Forest Research Institute, Vantaa, Finland (H. Henttonen); City of Haapavesi, Environmental Health Service, Haapavesi, Finland (L. Virtaluoto); 1 Current affiliation: European Centre for Disease Prevention and Control, Stockholm, Sweden.; 2 Current affiliation: Environmental Health Service, Nilsiä, Finland.

**Keywords:** Yersinia pseudotuberculosis, yersiniosis, foodborne outbreak, Finland, vegetable, carrots, reservoir, shrew, letter

**To the Editor:** Illness caused by *Yersinia pseudotuberculosis* is mainly characterized by fever and acute abdominal pain due to mesenteric lymphadenitis that mimics appendicitis. Secondary manifestations include erythema nodosum and reactive arthritis (*1*). Outbreaks have been reported in the Northern Hemisphere, including Canada (*2*,*3*), Japan (*4*), and Russia (*5*). Several community outbreaks have also been reported in Finland since 1982 (*1*,*6*–*9*). Only in a few of the outbreaks has the vector or source of the infection been identified. Recently, fresh produce, such as iceberg lettuce (*7*) and carrots (*9*), has been implicated by epidemiologic investigations as a source of infection, but mechanisms of contamination of fresh produce have remained unknown.

On April 8, 2004, the National Public Health Institute of Finland was informed of several cases of gastroenteritis in schoolchildren in 1 municipality in northern Finland. On April 13, 2004, stool samples from symptomatic schoolchildren confirmed *Y*. *pseudotuberculosis* infections. At the same time, an increase occurred in *Y*. *pseudotuberculosis* cases reported to the National Infectious Disease Register (NIDR) from other parts of the country. We conducted epidemiologic, microbiologic, trace-back, and environmental investigations to determine the source of the outbreak and the origin of contamination.

In the school outbreak, a survey concerning symptoms of gastrointestinal illness was conducted among all schoolchildren (7–18 years of age) and personnel (N = 900) of the 7 schools in the municipality. A case was defined as a laboratory-confirmed *Y*. *pseudotuberculosis* infection in a child or staff member who attended a school that received lunches from the school central kitchen, or as abdominal pain and fever, or erythema nodosum with illness onset from March 8 through March 28, 2004. Of respondents to the survey, 53 met the case definition. Among these respondents, *Y*. *pseudotuberculosis* was isolated from stool samples of 5 persons.

A case–control study was conducted to identify the source of infection; self-administered questionnaires asked about consumption of items on school menus from March 8 through March 26, 2004. For each of the 53 case-patients identified in the survey, 3 controls were selected from the same class; 39 cases and 107 controls were included the analysis. Univariate analysis showed that a vegetable mixture of carrots and white cabbage served on March 8 and a mixture of cucumber and white cabbage served on March 25 were associated with illness. Multivariate analysis showed that only the carrot–white cabbage mixture was associated with illness.

We also conducted a case–control study by mailing questionnaires to 37 persons with microbiologically confirmed *Y*. *pseudotuberculosis* infections reported to the NIDR from March 15 through May 7, 2004. These cases were from other parts of the country and were not associated with the school outbreak. For each case, 4 controls matched by age, sex, and municipality were randomly selected from the national population registry. Risk of illness increased with increased frequency of eating fresh carrots.

Carrots served in the school were traced back to the farm level. Samples checked included grated carrots, which were available from the school kitchen. The kitchen had received all vegetables from 1 fresh-food processing plant. Samples were taken from the carrot-peeling line, carrot-peeling leftovers, grated carrots, and other vegetable-processing lines at the plant. Carrots originated from only 2 farms, which were inspected, and samples were obtained for bacteriologic examination. Small mammals at the farms were caught in carrot fields and investigated microbiologically to identify the reservoir of *Y*. *pseudotuberculosis*. This bacterium was isolated from 1 environmental sample from the carrot-peeling line in the fresh-food processing plant, from spoiled carrots, from fluid draining from spoiled carrots, and from a pooled sample of common shrew (*Sorex araneus*) intestines from 1 farm.

Human and environmental isolates obtained were serotype O:1, subtype O:1b. Pulsed-field gel electrophoresis (PFGE) profiles of isolates from schoolchildren, fluid of spoiled carrots at the infected farm, and shrew intestines were indistinguishable. All 22 isolates from NIDR cases belonged to 2 PFGE genotypes. One genotype had a PFGE profile that was indistinguishable from the profile of the school outbreak isolates and the other genotype differed from these isolates by only 1 fragment.

Our study provides microbiologic and epidemiologic evidence that the school outbreak was caused by carrots contaminated at the production farm. We isolated a *Y*. *pseudotuberculosis* subtype from human patients that was indistinguishable from isolates from the implicated source and a potential animal reservoir. Although the association between shrews and carrots is uncertain, shrews may have been picked up with carrots by harvesting machinery and ended up dead in wooden storage frames with the carrots. If carrots become contaminated, long storage at cold temperatures favors growth of *Y*. *pseudotuberculosis* and may result in human infections. Further studies are needed to determine the mechanism of contamination and other natural reservoirs. After the outbreak, the Finnish Food Safety Authority recommended controlling contamination at the farm level by removing spoiled carrots and paying attention to any subsequent spoilage during handling procedures.
